# IMPROVING THE EFFECTIVENESS AND EFFICIENCY OF EVIDENCE PRODUCTION FOR HEALTH TECHNOLOGY ASSESSMENT

**DOI:** 10.1017/S0266462315000355

**Published:** 2015

**Authors:** Karen Facey, Chris Henshall, Laura Sampietro-Colom, Sarah Thomas

**Affiliations:** Evidence Based Health Policy Consultant; Health Economic Research Group, Brunel University London; HTA Unit, Hospital Clinic Barcelona; University of Southampton, Wessex Institutes.thomas@southampton.ac.uk

**Keywords:** Organizational innovation, Evidence-based health care, Decision making, Healthcare systems, Technology assessment

## Abstract

**Objectives:** Health Technology Assessment (HTA) needs to address the challenges posed by high cost, effective technologies, expedited regulatory approaches, and the opportunities provided by collaborative real-world evaluation of technologies. The Health Technology Assessment International (HTAi) Policy Forum met to consider these issues and the implications for evidence production to inform HTA. This paper shares their discussion to stimulate further debate.

**Methods:** A background paper, presentations, group discussions, and stakeholder role play at the 2015 HTAi Policy Forum meeting informed this paper.

**Results:** HTA has an important role to play in helping improve evidence production and ensuring that the health service is ready to adopt effective technologies. It needs to move from simply informing health system decisions to also working actively to align stakeholder expectations about realistic evidence requirements. Processes to support dialogue over the health technology life cycle need to be developed that are mindful of limited resources, operate across jurisdictions and learn from past processes. Collaborations between health technology developers and health systems in different countries should be encouraged to develop evidence that will inform decision making. New analytical techniques emerging for real-world data should be harnessed to support modeling for HTA.

**Conclusions:** A paradigm shift (to “Health Innovation System 2.0”) is suggested where HTA adopts a more central, proactive role to support alignment within and amongst stakeholders over the whole life cycle of the technology. This could help ensure that evidence production is better aligned with patient and health system needs and so is more effective and efficient.

The Health Technology Assessment International (HTAi) Policy Forum provides an opportunity for senior people from public and private sector organizations using health technology assessment (HTA) to support decisions or recommendations about product development and coverage to meet for strategic discussions about the present state of HTA, its development and implications for healthcare systems, industry, patients, and other stakeholders (1). Since 2007, the Policy Forum has published papers addressing issues about evidence generation, managing uncertainty and demonstration of value. In 2014, it concluded that evidence production needs to be optimized over the whole life cycle of the technology.

This influenced the choice of topic for the 2015 HTAi Policy Forum meeting, which combined two possible topics of “improving the effectiveness and efficiency of evidence production” with “adapting HTA to recent *trends* in technology development, HTA and health systems.” Given the breadth of the topics, a background paper (2) reviewed the “recent trends,” focusing on those that might impact HTA evidence production. This allowed discussion at the 2015 Policy Forum meeting to focus on improving evidence production in the light of these trends. This paper summarizes the discussions of the 2015 meeting and invites others involved in HTA to discuss and debate the issues raised.

## METHODS

The HTAi Policy Forum meeting was held February 8–10, 2015. Seventy-five people took part in the discussion, including Policy Forum and HTAi Board members and invited experts from a pharmaceutical industry association, a patient association, a medical device research collaboration and academia. Supplementary Table 1 lists all attendees.

Experts and members from different stakeholder groups gave opening presentations about key trends and issues relating to health technology evidence production. Then opportunities and challenges for improvements were discussed in separate workstreams for drugs, drugs with companion diagnostics, and therapeutic medical devices. (In this study, the term “drug” refers to medicines that are either pharmaceutical or biopharmaceutical medicinal products.)

The intention was to create models for evidence production and “stress test” models for drugs and devices using a “Fred Friendly” (3) discussion. The Fred Friendly discussion involved seven panelists playing the role of different stakeholders, with a moderator to draw out views about hard choices. A realistic but hypothetical situation was used that considered reimbursement of a new, potentially highly effective drug, and a new form of proton beam therapy, for fast-growing brain tumors. Plenary discussions then shared insights across all forms of technologies.

The meeting was conducted under the Chatham House Rule, whereby participants are free to share information obtained at the meeting, but they may not reveal the identity or affiliation of the person providing the information. This study summarizes the authors’ views of the key thoughts and suggestions emerging from the discussion at the meeting and the wider issues raised. While informed and strengthened by attendees’ comments on drafts, it is not a consensus statement from the meeting, nor can it be taken to represent the views of any of those attending the meeting or the organizations for which they work.

### Findings

The background paper outlined the current challenges faced by health systems and health technology developers and highlighted a range of interesting initiatives to support collaborative or real-world evaluation of promising technologies involving healthcare providers, payers, health technology developers, regulators, patient organizations, academics, and HTA organizations (2). Meeting participants agreed that this paper provided a good basis for discussion of the implications of these developments for evidence production.

The opening presentations and discussions in the meeting augmented the themes identified in the background paper, highlighting that we appear to be at a disruptive time in health service delivery, with several pressures but a range of exciting opportunities.

There are increasing financial pressures on all stakeholders and the desire to provide patients with earlier access to effective therapies where there is high unmet need. Therefore, issues of available evidence, uncertainty, and perceived value need to be balanced.

Waste in research needs to be reduced (4), and the value of research improved by focusing on relevant research questions, using appropriate designs and methods and delivering findings in accessible, unbiased publications (5).

There are exciting new developments in basic science that could lead to targeted, highly effective and curative treatments. Health systems are improving their electronic records and recording health outcomes, which can be analyzed using structured, sophisticated analyses in real-time (6). There are also new collaborative approaches between healthcare providers and technology developers to enable evaluation of technologies in the health system before adoption (7) or early in adoption to optimize use (8).

There is a need and an opportunity to harness these developments and improve the effectiveness and efficiency of evidence production for new health technologies to input to HTA and inform decision making. However, new approaches need to take account of the limited resources of all stakeholders and risks need to be shared to produce evidence that is sufficient for different decision makers. This requires elimination of unnecessary duplication and agreement on what evidence is needed, and when, recognizing that compromises may be required on all sides. To achieve this, there must be alignment of stakeholders, not just at the point of a decision about reimbursement, but earlier when decisions are made about which technologies to develop and what evidence to produce. Clinicians, managers, patients, and technology developers need to be involved to ensure that the process to a coverage decision is not only efficient but that it is also effective. To be effective, health services need to be organized to enable rapid and appropriate introduction of effective technologies and disinvestment of ineffective technologies.

This suggests an additional responsibility for HTA. The HTA function needs to act as a facilitator to help align objectives within and between stakeholder groups relating to the development and deployment of technologies. Some described this as HTA being a “broker,” bringing stakeholders together to create common understanding of issues and develop solutions. This would involve helping technology developers understand clinical and patient needs, evidence generation requirements, and limitations and helping health systems understand the potential and implications of new technologies and possible challenges of implementation. Others questioned whether this was a role for the HTA function alone, pointing to the importance of regulators, clinicians, managers, and patient organizations.

In practice, how this facilitator or broker function could and should be organized is likely to be dependent on the organization of health systems, available resources and a range of other factors, and is an important topic for debate. So within this context, break-out groups at the meeting discussed how health technology evidence production could be organized over the life cycle of a technology to make it more effective and efficient, and to consider the role of HTA in this context.

### Drugs

In the breakout discussions about the evidence development pathways for drugs, it was proposed that new approaches to clinical research and HTA are required, with more planned interaction between stakehholders before and during evidence production to align expectations and manage emerging risks.

In the preclinical phase and phase I, it may be too early for dialogue among stakeholders about specific evidence requirements. However, there could be helpful discussions about unmet needs in clinical practice, products in the pipeline, place in the clinical pathway, expected indications, potential size of market, anticipated benefits, regulatory approach, and forms of evidence likely to be required.

Early in phase II, dialogue may consider the potential population to be treated. Later in phase II, predictive models of the value of the technology are needed that seek to identify what evidence is needed, when. Specific issues around evidence production in phase III that are important for decisions on adoption should be discussed, including:

• which patients should be treated
• comparators (place in clinical pathway and comparators for direct or indirect comparisons)
• key outcomes (relevant for decision makers and patients) and method by which these can best be measured or estimated
• expected benefits in terms of effectiveness (including duration of benefit) and associated resource use
• generalizability of evidence
• organization of services to optimize delivery
• anticipated budget impact
• risk sharing and need for co-payments
• management of uncertainty.

After phase III, it will be important to report whether the evidence was produced according to plan. During regulatory approval and HTA and coverage decisions, dialogue is needed about how to manage uncertainty and fill evidence gaps in the light of the results achieved in the confirmatory trials.

Any systems that are established for postmarketing and postadoption evidence collection need to make it easy for clinicians to contribute (workflow, ease of use, incentives, etc); must have clear governance arrangements (tranparency, data access, appropriate analysis, feedback to contributors, funding, etc); and ensure efficient and flexible interactions among the necessary stakeholders.

Challenges that may arise include multiple views about the appropriate development pathway and evidence requirements among HTA agencies and regulators, so strategic choices will need to be made. Furthermore, drugs that are selected for adaptive or expidited regulatory pathways (9;10) may not fit the traditional phase I-, II-, III-related processes descibed above. Questions also remain about who will pay (both for the treatment and for its evaliuation), what incentives there will be for contributions at different stages in the technology lifecycle, and how HTA capacity can be built.

### Drugs with Companion Diagnostics

Discussion of evidence development for drugs with companion diagnostics noted that companion diagnostics are linked to the drug development life cycle, aiming to reduce uncertainty by identifying patients that will benefit most from the drug. Key questions arise about how companion diagnostics will fit into the clinical pathway, so in addition to the questions relevant for drugs, the following issues can be added:

• technical feasibility (screening potential patients to identify those best suited to the technology)
• performance of the test
• cutoff points for relevant subpopulations to determine treatment choices
• organization of healthcare testing systems (see EUnetHTA HTA Core Model® organizational domain)
• ethical questions (genetic issues: right to know, family issues, etc)
• regulatory requirements
• responsibility for payment (given siloed budgets).

Interestingly, it would seem that most of these issues are similar to the more general issues raised for drugs in phase I and so could be addressed early in the life cycle.

Given the low cost of diagnostics and the high cost of drugs, some members questioned whether such a complex HTA system is needed for companion diagnostics. Others pointed to the major impact that the proposed use of the diagnostic can have on feasibility, impact and value of the targeted therapy, and the consequent need to consider both the diagnostic and the drug in relation to one another in an HTA. A report by the UK Office of Health Economics suggests that a broad range of value elements and balanced analysis of diagnostic impacts is needed for companion diagnostics introduced at the launch of a new drug (11).

### Devices

Discussion emphasized that devices have a different context to drugs (12;13), including:

• development and use of many more medical devices than any other form of health technology
• rapid technology evolution and no obvious phase I, II, III delineations
• optimization of use is more complex (depending on user and setting)
• necessary infrastructure (capital outlay, software, training, etc)
• regulatory approval that does not require clinical evidence for all forms of devices
• health system use often determined at a hospital level and/or through a procurement, business case route, which is not informed by HTA.

Despite these differences, the evidence conundrum is similar, with a need for more efficient evidence production and assessment. This would ideally be achieved through a joint advice process discussing regulatory and payer needs across jurisdictions, to identify unmet needs, help design studies that are relevant for all key stakeholders, and identify how outcomes from different sources can be linked.

It was noted that some evidence requirements for devices can be identified early and involve purely scientific issues that can be documented in a protocol (real-world data, target populations, quality of life, resource use, costs, medium term effectiveness). Other topics require a different form of dialogue with stakeholders (e.g., feasability of randomized controlled trials and use of a device within a specific health system's organizational structure).

Different stakeholders will have differing views on what constitutes appropriate and useful evidence. Hence, dialogue for evidence production of devices needs to have a clear process, with:

• focus on the disruptive, innovative technologies with the aim of providing relevant evidence to health systems to optimize patient outcomes
• involvement of relevant experts (regulators, payers, clinical experts, patients, HTA, industry)
• a neutral convenor
• payer and industry infrastructure to enable effective engagement.

### Key Issues for All Technologies

In the plenary discussions, several key issues were identified of relevance to the optimal role and functioning of HTA across all classes of technologies.

### Key Role of HTA

HTA is undertaken to inform decisions, and it can and should inform a wider range of decisions than just those for reimbursement at the time of launch. It can also inform early decisions about whether to pursue development of a technology, and later decisions in clinical practice and health service organization about how best to adopt a technology and optimize its use. Hence, HTA should not be seen as an academic endeavor disconnected from technology development and healthcare delivery. Rather, it should be seen as providing a crucial interface between decision makers in health technology development, clinical practice, and health systems, bringing them, patients, and other key stakeholders together to agree on evidence requirements and then helping develop and evaluate that evidence to support informed decisions by all parties (14). It should help ensure that challenges in treating patients, evidence production, and technology use are understood and expectations are aligned to create realistic evidence requirements. This means HTA moves from simply being a passive assessment at one point in time, to also being an active facilitator of dialogue that informs evidence production, evidence-based decision making, and optimal technology use in the health system throughout the life cycle of the technology.

### Dialogue on Evidence, Impact, and Use

The provision of scientific advice on HTA requirements is seen as a key initiative to support effective and efficient evidence production (15;16). It cannot be binding on developers or HTA Agencies, but should lead to a culture where evidence requirements are not changed without good reason and evidence submissions provide what was agreed or justifcations for changes are explained. As scientific advice is confidential, it is difficult to obtain information about its impact. However, one Policy Forum member noted that parallel scientific advice meetings for five different products led to changes in the design of phase III development programs, helped gain alignment on an acceptable comparator, and influenced the studies undertaken and strategic decisions such as positioning of the product in the clinical pathway. In addition, the scientific advice process influenced company culture, helping to align regulatory affairs, health economics, and market access functions. This led to “access issues” and payer perspectives and needs having a much greater influence on important development decisions, alongside traditional clinical development influences. This view about the influence of the process on changing company culture was shared by other Forum members informally. However, the streamlined processes developed over 2 decades by regulators are not in place for many HTA agencies, and there remain challenges regarding divergent scientific advice both between regulatory and HTA agencies, and between HTA agencies in different jurisdictions.

More recently, the term “early dialogue” has been used for discussions of evidence that will inform HTA and coverage decisons (in addition to regulatory processes) (17). This has recognized that such processes should not be a one-way advice process from HTA or coverage bodies (or regulators) to technology developer, but should be a two-way dialogue and involve other relevant stakeholders such as patients and clinical experts. Dialogue should consider what is needed to demonstrate value in the national or regional system in the light of the practical limitations on evidence production in a global development program.

The workstreams in the Policy Forum meeting suggested an expansion of the role of this dialogue from being “early” (in advance of confirmatory trials) to being a series of discussions starting before many current dialogues and followed up at various points over the life cycle of the technology. It also noted that dialogue beyond the scientific aspects of clinical trial design can be helpful. Discussions about clinical pathways, unmet needs, and health system implementation may also be valuable. The authors suggest that this type of interaction might be better described by a system such as “dialogue on evidence, impact, and use.”

It is interesting to consider how HTA agencies could build capacity and develop efficient processes in collaboration with one another, with regulators and other key stakeholders to provide health technology developers with the broader range of advice at key points in the life cycle of drugs and devices proposed in this study. Such developments would be greatly facilitated if the outcomes and learnings from existing advice processes could be more widely shared and discussed, and this is starting to happen (18). Furthermore, consideration needs to be given to the development of more publicly available condition specific methodology guidelines to improve evidence production for HTA (19;20).

### Real-World Evaluations

Health systems are continuing to implement better administrative databases (21) and develop registries to optimize health technology use (22–24). The sophisticated techniques for data linkage and analysis that are now emerging and learnings from other sectors about the use of Big Data and rapid cycle analytics could help transform routine health system data into important evidence for HTA (25;26).

There seems to be a potential for better collaboration among health systems to work with technology developers to undertake international studies for evaluation of potentially innovative technologies to produce good quality pragmatic data efficiently that can inform HTA (27).

The Fred Friendly stakeholder role play confirmed the need for dialogue about evidence production for HTA, but showed that the form of evidence required will be intensely negotiated and dependent on several factors. The value of nontraditional data sources and study designs to augment randomized controlled trials and the value of patient involvement throughout the evidence production process are apparent. However, willingness to accept early evidence and the associated risks in expedited approvals will vary by health system and could be dependent on their control over data collection, their capability to encourage and restrict health technology use, and payment systems.

### Defining Value and Affordability

The discussion at the meeting stressed the need to revisit how value is judged and decisions on adoption are made. This issue has come into focus recently for very high cost technologies addressing high unmet needs and for high cost technologies addressing more common conditions, such as hepatitis C (28). There was recognition that, in most systems, budget impact has to be considered alongside value for the individual patient. There was also recognition that conventional measures of value such as incremental cost per QALY may be inappropriate or provide an insufficient basis for real-world policy and coverage decision making, but there is little agreement on what to do about this. For devices, additional issues such as purchasing processes, decentralized decisions, and the lack of HTA input to those decisions can impact the determination of value.

## CONCLUSION

HTA must be independent and objective, but it needs to develop more agile and adaptive processes that help to broker alignment among technology developers and health systems (including healthcare professionals and patients). The goal is to create a common understanding of health needs, the potential of new technologies to address needs and improve outcomes in the real world, and the need for health systems to adapt to realize those benefits. However, it needs to do this without increasing complexity, keeping it simple and comprehensible to the public.

This suggests that HTA needs to innovate and be prepared to play a more active role to influence evidence production and help facilitate dialogue among stakeholders to optimize technology development and use. Participants in the HTAi Policy Forum meeting referred to this new approach as HTA 2.0. But does this mean a new paradigm for the HTA function, or for the whole system?

The discussion at the HTAi Policy Forum clearly identified the need for fundamental re-thinking of a range of issues in addition to how evidence requirements can be agreed and addressed. These include the need to review concepts such as value and affordability, the need to consider costs and benefits more coherently across health and social care systems, and the need to manage services and systems so as to be more adaptable and responsive to promising new treatments. This requires engagement with payers, providers, patients, politicians, and the public to debate priorities, trade-offs, value, and acceptance of risk. It will need sophisticated architecture to deliver this and a champion at the highest policy level.

This could be described as a set of related paradigm shifts: HTA 2.0, Health System Organization and Management 2.0, Innovation System 2.0, Public Policy 2.0. Or it could be a single paradigm shift that embraces all these areas in a coherent manner: “Health Innovation System 2.0.” If it is the latter, how can all the key stakeholders be involved in discussion and development of it, and who could and should take the lead? These are some of the key issues and questions that the HTAi Policy Forum hopes its discussion will stimulate others to consider and debate, and to which it expects to return to at its 2016 meeting and elsewhere.

As a first step in this wider discussion, the issues raised in this paper were discussed at a panel session at the 2015 HTAi Annual Scientific Meeting in Oslo and publicly reported (29).

## POLICY IMPLICATIONS

• HTA needs to help facilitate dialogue among stakeholders over the life cycle of the technology to develop a shared understanding of evidence requirements to demonstrate value and enable rapid decisions about use of technologies.
• Technology developers can improve dialogue by explaining the scientific rationale underpinning the creation of a technology, providing a clear overview of information available, and posing clear questions to HTA and coverage bodies about future evidence requirements and approaches to value determination.
• Those involved in the wider health system such as patients, clinicians, and managers should be encouraged to contribute to this dialogue to help clarify unmet needs, quantify risks, determine value, and ensure rapid adoption of effective technologies.
• All stakeholders need to engage in a discussion about the development of this new collaborative approach to health innovation.

## Supplementary material

For supplementary material accompanying this paper visit http://dx.doi.org/10.1017/S026646231500035. click here to view supplementary material

## CONFLICTS OF INTEREST

The authors were funded by HTAi to organize and report on this meeting. In addition, Karen Facey and Chris Henshall have undertaken a range of other paid work relating to HTA with life sciences companies, consultancies, not-for-profit organizations and governments.
