# Supplementary material for: IMPROVING THE EFFECTIVENESS AND EFFICIENCY OF EVIDENCE PRODUCTION FOR HEALTH TECHNOLOGY ASSESSMENT
Source: Int J Technol Assess Health Care. 2015;31(4):201–6. doi: 10.1017/S0266462315000355 (PMC4697336; doi:10.1017/S0266462315000355)
Supplement: Supplementary file 1 [file S0266462315000355sup001.docx]

**Supplementary Table 1. Members of the HTAi Policy Forum, HTAi Policy Forum Committee, HTAi Board, HTAi Secretariat and invited speakers for the 8-10^th^ February 2015 HTAi Policy Forum meeting, Barcelona, Spain.**

| **Meeting Attendees** | |
| --- | --- |
| **Name** | **Organization** |
| Jeonghoon Ahn | National Evidence-based Healthcare Collaborating Agency (NECA), Korea |
| James Anderson | GlaxoSmithKline |
| Richard Bergstrom | European Federation of Pharmaceutical Industries and Associations |
| Meindert Boysen | National Institute for Health and Care Excellence (NICE), England |
| Andrew Bruce | AMGEN, Inc. |
| Lars Bruning | Bayer Pharma AG |
| Barbara Calvert | Abbott |
| Christophe Carbonel | Novartis Pharma AG |
| Christopher Chinn | GlaxoSmithKline |
| Americo Cicchetti | Univerita Cattolica del Sacro Cuore, Rome, Italy |
| Tammy Clifford | Canadian Agency for Drugs and Technologies (CADTH), Canada |
| Alexandra Clyde | Medtronic, Inc. |
| Elizabeth Cobbs | Merck |
| Nigel Cook | Novartis Pharma AG |
| Joseph Cook | Pfizer Inc |
| Anne D'Andon | French National Authority for Health (HAS), France |
| Andrew Dillon | National Institute for Health and Care Excellence (NICE), England |
| Karen Facey | HTAi Policy Forum Scientific Secretary (2015), Evidence based Health Policy Consultant |
| Katrine Fronsdal | Norwegian Knowledge Centre for the Health Services (NOKC), Norway |
| Sebastian Gaiser | St. Jude Medical |
| Eric Giesen | Bayer Pharma AG |
| Robert Giffin | Covidien |
| Wim Goetsch | National Healthcare Institute (ZINL), The Netherlands |
| Cliff Goodman | The Lewin Group |
| David Grainger | Eli Lilly and Company |
| Alicia Granados | Genzyme (a Sanofi Company) |
| Adrian Griffin | Johnson & Johnson |
| Jens Grueger | F. Hoffman-La Roche AG |
| Iñaki Gutiérrez-Ibarluzea | Basque Office for HTA (OSTEBA), Spain |
| Jean-Luc Harousseau | French National Authority for Health (HAS), France |
| Adam Heathfield | Pfizer Inc |
| Ansgar Hebborn | F. Hoffman-La Roche AG |
| Chris Henshall | HTAi Policy Forum Chair, Consultant |
| Kent Jancarik | EMD Serono, Inc. |
| Rabia Khaveci | Ankara Numune Training and Research Hospital, Turkey |
| Dell Kingsford Smith | Janssen Global Services |
| Marianne Klemp | Norwegian Knowledge Centre for the Health Services (NOKC), Norway |
| Sungkyu Lee | National Evidence-based Healthcare Collaborating Agency (NECA), Korea |
| Michael Lees | Bristol-Myers Squibb Co. |
| Leslie Levin | MaRS EXCITE, Canada |
| Jan Liliemark | Swedish Council on Health Technology Assessment (SBU), Sweden |
| Tae-Hwan Lim | National Evidence-based Healthcare Collaborating Agency (NECA), Korea |
| Carole Longson | National Institute for Health and Care Excellence (NICE), England |
| Eric Low | Myeloma UK, UK |
| Guy Maddern | University of Adelaide, Australia |
| Marjukka Makela | Finnish Office for HTA (FINOHTA), Finland |
| Deborah Marshall | University of Calgary, Canada |
| Newell McElwee | Merck |
| Clare McGrath | AstraZeneca |
| Francois Meyer | French National Authority for Health (HAS), France |
| Simona Montilla | Italian Medicines Agency (AIFA), Italy |
| James Murray | Eli Lilly and Company |
| Dan Ollendorf | Institute for Clinical and Economic Review (ICER), USA |
| Brian O'Rourke | Canadian Agency for Drugs and Technologies (CADTH), Canada |
| Britta Paschen | Merck Serono |
| Steve Pearson | Institute for Clinical and Economic Review (ICER), USA |
| Andres Pichon-Riviere | Institute for Clinical Effectiveness and Health Policy, Argentina |
| Johan Ponten | TLV (The Dental and Pharmaceutical Benefits Agency), Sweden |
| Sarah Puddicombe | University of Southampton, England |
| Herbert Riband | AMGEN, Inc. |
| Milena Richter | Genzyme (a Sanofi Company) |
| Adrienne Ross | Bristol-Myers Squibb Co. |
| Murray Ross | Kaiser Permanente |
| Alric Ruether | Institute for Quality and Efficiency in Health Care (IQWiG), Germany |
| Laura Sampietro-Colom | HTAi Policy Forum Vice Chair, Hospital Clinic Barcelona, Spain |
| Chris Sargent | Health Technology Assessment International |
| Linda van Sasse | National Healthcare Institute (ZINL), The Netherlands |
| Sebastian Schneeweiss | Harvard Medical School, USA |
| Markus Siebert | St. Jude Medical |
| Mitchell Sugarman | Medtronic, Inc. |
| Sarah Thomas | HTAi Policy Forum Scientific Programme Manager, University of Southampton, England |
| Sean Tunis | Centre for Medical Technology Policy (CMTP), USA |
| Sophie Soderholm Werko | Swedish Council for Technology Assessment in Healthcare (SBU), Sweden |
| **HTAi Policy Forum Committee** | |
| **Name** | **Role and Organization** |
| Barbara Calvert | HTAi Policy Forum Committee member, Abbott |
| Laura Sampietro-Colom | HTAi Policy Forum Vice Chair, Hospital Clinic Barcelona, Spain |
| Joseph Cook | HTAi Policy Forum Committee member, Pfizer Inc |
| Karen Facey | HTAi Policy Forum Scientific Secretary (2015), Evidence based Health Policy Consultant |
| Ansgar Hebborn | HTAi Policy Forum Committee member, F. Hoffman-La Roche AG |
| Chris Henshall | HTAi Policy Forum Chair, Consultant |
| Carole Longson | HTAi Policy Forum Committee member, National Institute for Health and Care Excellence (NICE), England |
| Guy Maddern | HTAi Policy Forum Committee member, University of Adelaide, Australia |
| Lloyd Sansom | HTAi Policy Forum Committee member, University of South Australia, Australia |
